# Study of a common azo food dye in mice model: Toxicity reports and its relation to carcinogenicity

**DOI:** 10.1002/fsn3.906

**Published:** 2019-01-29

**Authors:** Md. Sajib Al Reza, Md. Mahmudul Hasan, Md. Kamruzzaman, Md. Imam Hossain, Md. Abu Zubair, Luthfunnesa Bari, Md. Zainul Abedin, Md. Abu Reza, Khandaker Md. Khalid‐Bin‐Ferdaus, Kazi Md. Faisal Haque, Khairul Islam, Mahtab Uddin Ahmed, Md. Khaled Hossain

**Affiliations:** ^1^ Department of Food Technology and Nutritional Science Faculty of Life Science Mawlana Bhashani Science and Technology University Tangail Bangladesh; ^2^ Protein Science Lab Department of Genetic Engineering and Biotechnology Faculty of Science University of Rajshahi Rajshahi Bangladesh; ^3^ Department of Biotechnology and Genetic Engineering Faculty of Life Science Mawlana Bhashani Science and Technology University Tangail Bangladesh; ^4^ Department of Biochemistry and Molecular Biology Faculty of Life Science Mawlana Bhashani Science and Technology University Tangail Bangladesh; ^5^ Biochemistry & Cell Biology Lab Bangladesh University of Health Sciences Dhaka Bangladesh; ^6^ Department of Biochemistry and molecular biology Faculty of Science University of Rajshahi Rajshahi Bangladesh

**Keywords:** carmoisine, gene expression, liver, synthetic food dye, toxicity

## Abstract

This study was conducted to evaluate the toxic effects of an azo dye carmoisine widely used in foods and to investigate its relation to carcinogenicity. Carmoisine administered into mice orally in four different doses as control, low, medium, and high equivalent to 0, 4, 200, and 400 mg/kg bw, respectively, for 120 days. The key toxicological endpoint was observed including animal body weight, organ weights, hematology, biochemistry, and molecular biology assessment. The body weights of medium‐ and high‐dose carmoisine‐treated mice group were significantly decreased as compared to the control mice group. Platelet, white blood cell and monocyte counts of treated group were considerably higher, while Hb and red blood cell counts were drastically lower than the control group. The biochemical parameters such as serum alanine aminotransferase, aspartate aminotransferase, alkaline phosphatase, total protein, globulin, urea, and creatinine level were significantly increased, while serum cholesterol level was decreased after treatment as compared to the control. RT‐PCR results showed that expression of Bcl‐x and PARP gene was intensively increased, whereas expression of p^53^ gene was decreased in the mouse liver tissues treated with carmoisine. This study revealed that high‐dose (400 mg/kg bw) treatment of carmoisine was attributable to renal failure and hepatotoxicity. It also would be suspected as a culprit for liver oncogenesis.

## 1 INTRODUCTION

Color additives have been used by the Ancient Egyptians. Historians estimated that colored food appeared by 1500 B.C. Now, the food manufacturers deem color as a vital criterion for food choice; thus, synthetic colorants are now being widely used due to their coloring properties, uniformity, stability, low cost, and availability in many hues, which overall increase the esthetic quality (Mpountoukas et al., 2010). The literature records estimate the production of synthetic colorants to be 8,000,000 tons per annum (Revankar & Lele, 2007).

Below the acceptable daily intake (ADI) limit, the intake of artificial colorants seems to be safe. However, consumption of high concentrations of these artificial colorants may result in many ailments especially in children due to their low body weights (Dixit, Purshottam, Khanna, & Das, 2011). Children are the major consumers of the colored food in developing countries, and thus, they are in the high risk because most often they exceed prescribed ADIs (Husain et al., 2006; Rao, Bhat, Sudershan, Krishna, & Naidu, 2004). An average of 48% food manufacturers adheres to the maximum allowable limit of 100 mg/kg, whereas the remaining 52% use excess of the prescribed limit. This is an alarming issue in the most developing countries. As for example, the use of a blend of sunset yellow FCF and tartrazine in India has exceeded the prescribed limit by 4.5‐ to 25.7‐fold in food products. Only carmoisine contributes up to 55% to the average intake of colored food products. So the intake of synthetic food colorants exceeds their respective ADI limits in almost all age groups (Dixit, Purshottam, Gupta, Khanna, & Das, 2010; Dixit et al., 2011). Thus, exposure to excessive colorants to the population may pose a serious health risk.

Carmoisine is a red to maroon food colorant of azo dye group with an aromatic structure. It is approved by US Food and Drug Administration (FDA). It usually exists as a disodium salt and is widely used in food, agrochemical, cosmetics, textile, paper, and pharmaceutical industries (Snehalatha, Ravikumar, Joe, Sekar, & Jayakumar, 2009). It is used in various food items such as chocolate, jams, jellies, Swiss roll, marzipan, preserves, yogurts, blancmange, breadcrumbs, and cheesecake mixes. Carmoisine is also used in oraldene mouthwash as a colorant (Amin, Hameid, & Elsttar, 2010). Owing to the appearance of an azo group, carmoisine can be reduced by intestinal microorganisms in vivo to an aromatic amine, which is highly sensitized. Sulfanilic acid is the chief metabolite of carmoisine (Chung, Stevens, & Cerniglia, 1992). Nitrite compounds present in various foods after combining with certain amino acids transform into N‐nitroso compounds or nitrosamines, and these are carcinogenic (Grosse et al., 2006). International Agency for Research on Cancer classifies azo dyes like carmoisine as category 3 carcinogens (Refat et al., 2008).

It has been reported that synthetic food colors induced behavioral changes in children, for example, hyperactivity (McCann et al., 2007). The individual response depends not only on dose, age, gender, nutritional status, and genetic factors but also on long‐term exposure to low doses (Amin et al., 2010; Sasaki et al., 2002). Carmoisine alters biochemical markers in vital organs even at low doses and reduces the helical composition by changing the secondary structure of hemoglobin (Hb; Basu & Kumar, 2015).

Azo dyes can induce chromosomal aberrations in mammalian cells (Patterson & Butler, 1982) and cause DNA damage in the colon of mice (Sasaki et al., 2002). Carmoisine shows positive for genotoxicity and increases concentrations of RNA (not DNA) in rat liver cell homogenates (Aboel‐Zahab et al., 1997; Ali, Al‐Ghor, Sharaf, Mekkawy, & Montaser, 1998; El‐Saadany, 1991). It also induces moderate conformational perturbations in the secondary structure of DNA after binding with DNA in media components containing glucose leading to the formation of the toxic component(s), which completely inhibited the growth of *Tetrahymena pyriformis* (Basu & Kumar, 2014; Marathe, Adhikari, Netrawali, & Nair, 1993). Expression of some fuel metabolism genes, for example, PPAR‐alpha, ACo‐A and CPT‐1, shows down‐regulation, which indicates that carmoisine may decrease the fuel metabolism in liver (Montaser & Alkafafy, 2013). Hydrophobic azo dyes are unsafe causing tumors in the liver and urinary bladder of rats (Golka, Kopps, & Myslak, 2004).

Due to the increasing and unregulated use by food manufacturers in many underdeveloped and developing countries, carmoisine consumptions exceed ADI level. Therefore, this study was aimed to investigate the potential toxic effects of carmoisine in mice model administering oral dose in their feed over the course of 120 days and to correlate such effects to develop carcinogenicity.

## 2 MATERIALS AND METHODS

### 2.1 Test article and animals

Carmoisine (E122) or Food Red 3 was purchased from Millennium Chemical Company (Dhaka, Bangladesh), and it was manufactured by Sun Food Tech. (Rajasthan, India). Swiss albino male mice of approximately 5 weeks’ healthy adults were purchased from ICDDR, B (International Centre for Diarrhoeal Disease Research, Bangladesh). After selection, randomly the animals were housed in clean polycarbonate cages with steel wire tops and corncob bedding. They were acclimated and maintained with 12‐h:12‐h dark–light cycle with available supply of distilled water and feed for 1 week. Animals were maintained in accordance with the “Guide for care and use of laboratory animals” (National Research Council, 1996).

### 2.2 Experimental design

This experiment was designed for 120 days, and details are shown in Table 1. At the starting of the experiment, the animals were approximately 6 weeks of age. They were divided into four equal groups named control, low, medium, and high carmoisine‐treated group, and each group contained 10 mice using tail tattoo as identification mark. Normal mice diet was prepared by homogeneous mixing of commercially available food ingredients. Carmoisine was mixed at the doses of 0, 4 (equivalent to ADI), 200 mg/kg bw per day (50‐fold ADI), and 400 mg/kg bw per day (100‐fold ADI) with normal diet for control, low, medium, and high group of mice, respectively. The general health, mortality, and any sign of sickness of animals were checked every day, and animal body weights were recorded individually once in a week. The daily food consumption per animal was calculated in order to determine feed efficacy ratio.

**Table 1:** Experimental design

Dose name	Target dosage level (mg/kg bw per day)	Number of animals (male)	Dose material	Anesthesia schedule (days/no of animals)
Control	0	10	Standard diet	120/10
Low dose	4	10	Standard diet + color	120/10
Medium dose	200	10	Standard diet + color	120/10
High dose	400	10	Standard diet + color	120/10

### 2.3 Sample collection and serum preparation

The mice were euthanized with diethyl ether after fasted overnight (12 hr). The blood samples were collected from thoracic aorta and were kept about 20 min at a room temperature for coagulation. Then, blood samples were centrifuged at 1600 g for 15 min at 4°C, and serum was drawn off and stored at −80°C for biochemical analysis. For hematology analysis, blood samples were kept in microcollection tubes containing potassium EDTA. After necropsy, selected organs were excised and rinsed in a chilled saline solution, and then blotted on filter paper and weighed separately to calculate relative organ weight. For molecular biology analysis, fresh tissues from each of the selected organs were used.

### 2.4 Hematology analysis

The hematological parameters, red blood cell (RBC), Hb, hematocrit, mean capsular volume, mean cell Hb concentration, mean cell Hb, platelets (PLT), white blood cell count (WBC), red blood cell distribution width, mean platelet volume (MPV), nonsegmented neutrophils, segmented neutrophils, lymphocytes, monocytes (MON), eosinophils, and basophils, were analyzed using a Sysmex XT‐2000i automated hematology analyzer (TOA Medical Electronics Co., Ltd., Hyogo, Japan).

### 2.5 Biochemical analysis

The semi‐automatic photometer analyzer (Humalyzer 3000, Wiesbaden, Germany) was used to measure alanine aminotransferase (ALT), aspartate aminotransferase (AST), alkaline phosphatase (ALP), total bilirubin (TBIL), total protein (TP), albumin (ALB), globulin (GLOB), urea (URE), creatinine (CRE), cholesterol (CHO), and triglycerides (TG) using commercially available reagents kits according to the manufacturer's protocol.

### 2.6 Organ weights

Various organs of mice were examined macroscopically during necropsy. Organ weights were recorded as absolute weights to calculate the relative organ weight (percentage of body weight).

### 2.7 RNA isolation

Three milligrams of fresh tissues from control and high‐dose carmoisine‐treated mice was thoroughly homogenized before RNA extraction using a Precellys 24 homogenizer (Bertin Technologies, Montigny‐le‐Bretonneux, France). Total RNA from tissues was extracted using RNA simple total kit (Tiangen Biotech Co. Ltd., Beijing, China) according to the manufacturer's instructions and was eluted in 50 μl RNase‐free H_2_O. On‐column DNase digestion was carried out to eliminate the carryover of DNA with the extracted RNA. RNA was then stored at −80°C for downstream applications. Amount of RNA concentration was assessed with the NanoDrop ND‐1000 UV–Vis Spectrophotometer (NanoDrop Technologies, Wilmington, DE, USA). The 260/280 ratios of RNA extracted were 1.88 and 1.85 in liver from control and treated mice, respectively. Integrity of the RNA quality was also checked by visualizing the 18S and 28S ribosomal RNA bands in 1.0% RNase‐free agarose gels.

### 2.8 Reverse transcription–polymerase chain reaction (RT‐PCR)

Expression levels of four target genes were measured semi‐quantitatively using RT‐PCR technique. RNA samples were first reverse‐transcribed into cDNA using TIANScript M‐MLV reverse transcriptase (Tiangen Biotech Co. Ltd., Beijing, China). Three micrograms of RNA was reverse‐transcribed into cDNA in a final volume of 20 μl containing 2 μl of 10 mM oligo (dT) primer 2 μl of 10 mM dNTPs mix and 1 μl TIANScript M‐MLV reverse transcriptase. Reverse transcription was performed at 42°C for 50 min by using oligo (dT) primers (10 μM). cDNAs were stored at −20°C until usage. PCR was performed as 25 μl reaction volume using 1 μg cDNA as template.

Expression of Bcl‐x, PARP, and p^53^ genes was studied, and GAPDH gene was used as control. Each PCR mixture consisted of 1× *Taq* DNA polymerase buffer, 0.5 μl of each primer from 10 mM stock, 0.5 μl of dNTPs mix (10 mM each), and 0.25 U of Tiangen *Taq* platinum DNA polymerase (Tiangen Biotech Co. Ltd., Beijing, China) in a Astec‐482 (Astec, Japan) thermal cycler. The cycling condition was initial PCR activation step of 6 min at 94°C, followed by 35 cycles of a 45‐s denaturation step at 94°C, a 45‐s annealing step at 52°C (for GAPDH and PARP), 60‐s synthesis step at 72°C, and a final extension of 72°C for 10 min. The annealing temperature for Bcl‐x and p^53^ was 55°C instead of 52°C. Upon completion of the reaction, 5 μl of the PCR products was analyzed by running it in 1% agarose gel stained with ethidium bromide. Tiangen 1Kb plus DNA ladder (Tiangen Biotech Co. Ltd., Beijing, China) was used as standard. PCR products were visualized at 302 nm using the Protein Simple gel documentation system ATI26D (Taiwan). Primer sequences used for the PCR to check the transcriptional levels of the target genes are shown in Table 2.

**Table 2:** Primer sequences and amplification sizes of the RT‐PCR assays used in the current experiment

Gene	Marker	Tissue	Primer sequence (5′‐3′)	Amplification size (bp)
GAPDH	House‐keeping gene	Liver	Fwd‐GTGGAAGGACTCATGACCACAG Rev‐CTGGTGCTCAGTGTAGCCCAG	350
Bcl‐x	Apoptosis	Liver	Fwd‐CTAGAATTCAAATGTCTCAGAGCAACCG Rev‐CAGAATTCAGGCCTGAACAATCGGTATCT	700
PARP	DNA repair gene	Liver	Fwd‐AGGCCCTAAAGGCTCAGAAT Rev‐CTAGGTTTCTGTGTCTTGAC	100
P^53^	Tumor suppressor	Liver	Fwd‐GCGTCTTAGAGACAGTTGACT Rev‐GGATAGGTCGGCGGTTCATGC	550

### 2.9 Statistical analysis

Statistical analyses were performed for body weights, body weight gains, hematology, biochemistry, and organ weights. Each data set was subjected to a statistical decision tree. To check the homogeneity, the variance data sets were initially analyzed using Levene's test (Levene, 1960), which was followed by the Shapiro–Wilk test to perform normality analysis (Royston, 1982). To reject the null hypothesis, a *p* < 0.001 level of significance was used for each test. The “classical” statistical testing procedure consisting of parametric and nonparametric method was used to compare groups. When both Levene's test and the Shapiro–Wilk test were not significant, a parametric method of a single‐factor analysis of variance (ANOVA) was applied where animal grouping was considered as the factor, using a minimum significance level of *p* < 0.05 (Gad & Weil, 1989). If the parametric ANOVA was significant at *p* < 0.05, post hoc Dunnett's test was used to identify statistically significant differences at *p* < 0.05 between the control group and each carmoisine‐treated group (Dunnett, 1955). When either Levene's test or the Shapiro–Wilk test was significant, nonparametric contrast testing method was applied. As a nonparametric method, the Kruskal–Wallis ANOVA was applied where animal grouping was considered as the factor, using a minimum significance level of *p* < 0.05 (Siegel, 1956). If the Kruskal–Wallis nonparametric ANOVA was significant at *p* < 0.05, Bonferroni multiple comparisons were applied to determine statistically significant differences between the control group and each carmoisine‐treated group using a minimum significance level of *p* < 0.05 (Glantz, Slinker, & Neilands, 1990).

## 3 RESULTS

In this paper, the term “significant” indicates a statistically significant change when compared with corresponding controls.

### 3.1 Visual observation of mice

No mortality was observed throughout the experiment. All visual findings were observed either incidental (i.e., thin appearance, animal found low food intake, staining of hair in the anogenital region); commonly observed in laboratory mice (i.e., hair loss due to friction against cage, scabs, reddish brown or soft stool, raised areas); or cage‐related accidental injuries (i.e., red material in cage, swelling, dark material around the nose and/or eyes). Such observations were rarely seen and not related to administration of carmoisine. The feces of mice fed at medium and high doses were reddish and purple. Yellow‐colored or reddish‐brown‐colored urine was noted at all treatment levels within one week of the initiation of the treatment. Low motor activities were observed in high‐dose group within 9, 11, and 16 weeks of the study.

### 3.2 Growth pattern of treated mice

**Figure 1:**
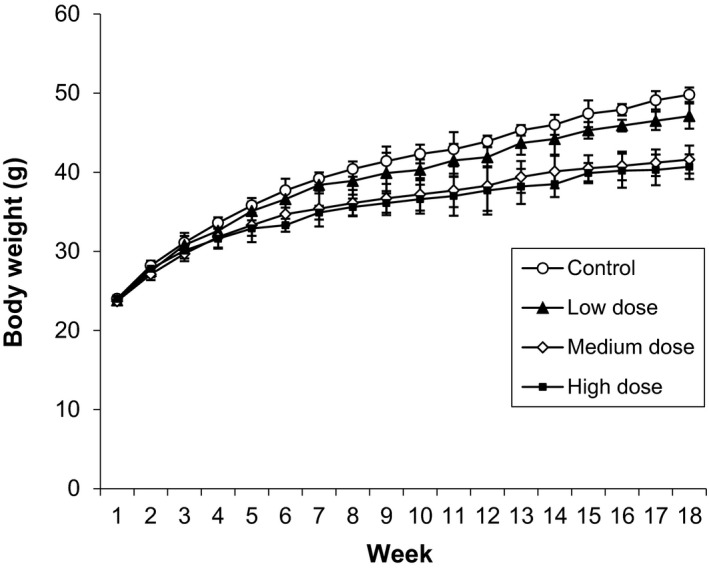
Growth curves of carmoisine‐treated male Swiss albino mice

Growth curves of the animals during the course of the study are illustrated in Figure 1. The mice fed with medium and high dose of carmoisine in the diet have significantly (*p *<* *0.05) lower body weights from week 6 to the end of the experiment.

Weekly body weight gains are presented in Table 3. The body weight gains of the medium‐dose‐treated group were significantly lower than the control group during 0–1 and 13–14 weeks. Similar trends were observed for high‐dose‐treated group during weeks 3–4, 4–5, and 15–16 correspondingly. At the end of the study, the weight gains of medium‐ and high‐dose‐treated mice groups were 17.9 and 16.7 g, respectively, as compared to the control group (25.8 g) (Table 4). Such findings might be considered as adverse effects of the magnitude of the difference and dose dependency of the carmoisine administration among the different groups.

**Table 3:** Body weight gains of male Swiss albino mice fed carmoisine (g/wk)

Week	Treatment group			
	Control	Low dose	Medium dose	High dose
0–1	4.2 ± 0.4^a^	3.7 ± 0.6	3.4 ± 0.6^b^	3.8 ± 0.6
1–2	2.9 ± 0.5	3.3 ± 0.8	2.6 ± 0.5	2.3 ± 1.0
2–3	2.5 ± 0.5	1.8 ± 0.9	2.1 ± 0.7	1.5 ± 1.0
3–4	2.2 ± 0.4	2.5 ± 0.9	1.5 ± 0.8	1.3 ± 0.4^b^
4–5	1.9 ± 0.5	1.5 ± 0.7	1.4 ± 0.5	0.4 ± 0.5^c^
5–6	1.5 ± 0.7	1.8 ± 0.6	0.7 ± 0.9	1.6 ± 0.6
6–7	1.2 ± 0.4	0.5 ± 0.5	0.7 ± 0.6	0.7 ± 0.6
7–8	1.0 ± 0.6	1.0 ± 0.6	0.6 ± 0.6	0.5 ± 1.0
8–9	0.9 ± 0.5	0.4 ± 0.8	0.5 ± 0.9	0.5 ± 0.8
9–10	0.6 ± 0.6	1.2 ± 0.4	0.3 ± 0.8	0.4 ± 0.9
10–11	1.0 ± 0.8	0.4 ± 0.5	0.6 ± 0.5	0.7 ± 0.4
11–12	1.4 ± 0.5	1.8 ± 0.7	1.1 ± 0.7	0.5 ± 1.0
12–13	0.7 ± 0.6	0.5 ± 0.5	0.7 ± 0.4	0.3 ± 0.9
13–14	1.4 ± 0.5	1.1 ± 0.7	0.4 ± 0.6^c^	1.4 ± 0.8
14–15	0.5 ± 0.7	0.6 ± 0.8	0.3 ± 0.8	0.3 ± 0.4
15–16	1.2 ± 0.4	0.6 ± 0.6	0.4 ± 0.8	0.1 ± 0.9^c^
16–17	0.7 ± 0.6	0.6 ± 1.5	0.4 ± 0.6	0.6 ± 0.8

a Values are expressed as mean ± *SD*.

b Significantly different from control group at *p* < 0.05.

c Significantly different from control group at *p* < 0.01.

**Table 4:** Body weight and feed consumption of mice

Dose level (mg/kg bw per day)	Mean body weight (g)			Percent change over control	Feed consumption	
	Initial	Final	Body weight gains (g)		Total feed intake (g) in 120 days	Feed efficiency ratio
Control	24.0 ± 0.47	49.8 ± 0.91	25.8		576	0.044
Low	23.8 ± 0.42	47.1 ± 1.59	23.3	95	612	0.038
Medium	23.7 ± 0.48	41.6 ± 1.77^a^	17.9	84	600	0.03
High	24.0 ± 0.31	40.7 ± 1.56^a^	16.7	82	594	0.028

Notes

Weights are given as mean ± *SD*. Body weight gains and total feed intake are given as mean.

a Significantly different from control group at *p* < 0.05.

Table 4 demonstrates an increase in total feed intake in all treated groups compared with the control group. The differences in feed intake among groups were relatively minor, and there were no clear test article‐related effects on feed consumption and feed efficacy.

### 3.3 Hematological findings

The results of hematological examinations by the end of the study are summarized in Table 5. A minimal decrease in RBC was noticed in high‐dose group. Significant decrease in Hb concentration was observed in medium‐ and high‐dose groups. Platelets and white blood cells were significantly increased in medium‐ and high‐dose groups than in control group. A significant increase has been shown in percentage of monocyte in high‐dose group as compared to control group.

**Table 5:** Hematological data for male Swiss albino mice given carmoisine for 120 days

Parameter	Unit	Treatment group			
		Control	Low dose	Medium dose	High dose
RBC	×10^12^/L	8.5 ± 0.2^a^	8.7 ± 0.5	8.5 ± 0.6	7.8 ± 0.7^b^
Hb	g/dl	15.1 ± 0.3	15.4 ± 0.6	14.0 ± 0.7^c^	14.3 ± 0.9^b^
HCT	%	41.5 ± 1.3	41.4 ± 2.1	42.5 ± 2.2	40.1 ± 1.7
MCV	fl	51.6 ± 0.3	51.7 ± 0.4	52.0 ± 0.6	51.7 ± 0.3
MCH	pg	16.8 ± 0.4	17.3 ± 0.6	16.4 ± 0.4	16.3 ± 0.3
MCHC	b/dl	30.8 ± 0.4	30.6 ± 0.5	31.4 ± 0.8	31.0 ± 0.5
PLT	10^9^/l	753.7 ± 63.6	768.1 ± 82.7	947.6 ± 102.0^c^	1061.8 ± 91.8^c^
WBC	10^3^/μl	11.3 ± 2.0	11.8 ± 2.4	13.9 ± 2.6^b^	14.2 ± 2.1^b^
RDW	%	17.6 ± 0.9	17.4 ± 1.0	17.6 ± 0.6	17.9 ± 0.6
MPV	fl	7.3 ± 0.9	7.3 ± 1.1	6.9 ± 1.0	6.7 ± 0.8
Differential cell count					
Nonseg. neut.	%	0.0 ± 0.0	0.0 ± 0.0	0.0 ± 0.0	0.0 ± 0.0
Seg. neut.	%	18.2 ± 6.6	17.0 ± 6.2	17.7 ± 4.9	19.4 ± 4.7
LYM	%	78.5 ± 7.3	80.2 ± 6.1	79.7 ± 5.3	77.1 ± 5.6
MON	%	1.3 ± 0.7	1.5 ± 1.5	1.8 ± 0.8	2.6 ± 1.2^b^
EOS	%	1.2 ± 0.6	1.0 ± 0.6	0.8 ± 0.3	0.9 ± 0.3
BAS	%	0.3 ± 0.2	0.3 ± 0.2	0.2 ± 0.1	0.2 ± 0.2

BAS, basophils; EOS, eosinophils; Hb, hemoglobin; HCT, hematocrit; LYM, lymphocytes; MCH, mean cell hemoglobin; MCHC, mean cell hemoglobin concentration; MCV, mean corpuscular volume; MON, monocytes; MPV, mean platelet volume; Nonseg. neut., nonsegmented neutrophils; PLT, platelets; RBC, red blood cells; RDW, red cell distribution width; Seg.neut., segmented neutrophils; WBC, white blood cells.

a Values are expressed as mean ± *SD*.

b Significantly different from control group at *p* < 0.05.

c Significantly different from control group at *p* < 0.01.

### 3.4 Biochemical findings

Blood serum biochemical data of the control and carmoisine‐treated mice are summarized in Tables 6 and 7. Treatment‐related effects on serum chemistry consisted of changes in ALT, AST, TP, GLOB, URE, and CRE in all dose groups in an exposure‐related manner. Statistically significant increments were observed for ALT, AST, TP, URE, and CRE in medium‐ and high‐dose groups and for GLOB in high‐dose group. Evidence of hepatocellular toxicity was manifested by significant increases in ALP activity in medium‐ and high‐dose groups but in a lack of dose dependency. Increased TBIL concentration that was found in group exposed to high dose of carmoisine also supported the possibility of hepatic toxicity. However, minimal but significant decrease in CHO value was observed in mice treated with high‐dose carmoisine.

**Table 6:** Analysis of liver function test of carmoisine‐treated mice

Parameter	Unit	Treatment group			
		Control	Low dose	Medium dose	High dose
ALT	U/L	32.12 ± 3.96^b^	35.65 ± 2.83	41.35 ± 3.75^b^	46.82 ± 4.53^b^
AST	U/L	58.46 ± 4.31	59.88 ± 4.01	65.26 ± 7.22^a^	71.53 ± 5.29^b^
ALP	U/L	87.67 ± 7.30	91.21 ± 7.55	113.63 ± 14.65^b^	99.48 ± 8.87^a^
TBIL	mg/dl	1.14 ± 0.16	0.90 ± 0.11	1.55 ± 0.40	1.89 ± 0.47^b^
TP	g/dl	6.48 ± 0.27	6.51 ± 0.24	6.83 ± 0.33^a^	7.32 ± 0.19^b^
ALB	g/dl	3.54 ± 0.71	3.50 ± 1.00	3.71 ± 0.74	3.67 ± 0.70
GLOB	g/dl	1.67 ± 0.11	1.70 ± 0.18	1.82 ± 0.15	2.15 ± 0.12^b^

ALB, albumin; ALP, alkaline phosphatase; ALT, alanine aminotransferase; AST, aspartate aminotransferase; GLOB, globulin; TBIL, total bilirubin; TP, total protein.

Values are expressed as mean ± *SD*.

a Significantly different from control group at *p* < 0.05.

b Significantly different from control group at *p* < 0.01.

**Table 7:** Analysis of kidney function tests and lipid profile of carmoisine‐treated mice

Parameter	Unit	Treatment group			
		Control	Low dose	Medium dose	High dose
URE	mg/dl	20.37 ± 0.68^a^	21.42 ± 0.91	26.70 ± 1.15**	37.12 ± 2.50**
CRE	mg/dl	0.86 ± 0.02	0.89 ± 0.05	0.91 ± 0.04*	1.45 ± 0.02**
CHO	mg/dl	133.27 ± 8.71	131.76 ± 6.84	116.73 ± 13.05**	121.69 ± 10.54*
TG	mg/dl	129.62 ± 6.56	132.50 ± 11.56	140.59 ± 12.84	138.49 ± 8.40

CRE, creatinine; CHO, cholesterol; TG, triglycerides; URE, urea.

Values are expressed as mean ± *SD*.

Significantly different from control group at *p* < 0.05.

Significantly different from control group at *p* < 0.01.

### 3.5 Macroscopic examination of organs

There were few gross macroscopic findings observed, and none of the findings were considered to be carmoisine‐related without histopathological examination. A variety of common inflammatory lesions were observed in liver and kidney tissues macroscopically. As these lesions were observed among both the control and treated mice groups, therefore, it might be considered as incidental and may be observed in this strain and age of mice naturally.

Table 8 shows the weight of whole body and different organs in percentage. Liver weight of mice exposed to medium and high dose was significantly decreased as compared to the control. Heart and kidney weights of the high‐dose‐treated mice group were also significantly lower than control group. The brain weights of mice exposed to medium dose were significantly higher when compared to controls.

**Table 8:** The weight (%) of whole body and different organs of carmoisine‐treated mice

Treatment group				
Organ	Control	Low dose	Medium dose	High dose
Whole body weight (final)	49.8 ± 0.91^a^	47.1 ± 1.59	41.6 ± 1.77*	40.7 ± 1.56*
Brain	0.92 ± 0.02	0.93 ± 0.03	0.96 ± 0.02*	0.94 ± 0.02
Heart	0.45 ± 0.03	0.44 ± 0.01	0.43 ± 0.01	0.42 ± 0.01*
Lung	1.02 ± 0.03	1.01 ± 0.02	1.03 ± 0.03	0.99 ± 0.03
Spleen	0.68 ± 0.02	0.68 ± 0.02	0.67 ± 0.04	0.66 ± 0.02
Liver	5.62 ± 0.53	5.62 ± 0.51	5.04 ± 0.20**	5.01 ± 0.20**
Stomach	1.75 ± 0.02	1.75 ± 0.03	1.79 ± 0.07	1.71 ± 0.06
Kidneys	0.70 ± 0.01	0.70 ± 0.01	0.69 ± 0.02	0.67 ± 0.02**

Values are expressed as mean ± *SD*.

Significantly different from control group at *p* < 0.05.

Significantly different from control group at *p* < 0.01.

### 3.6 Molecular analysis with RT‐PCR

**Figure 2:**
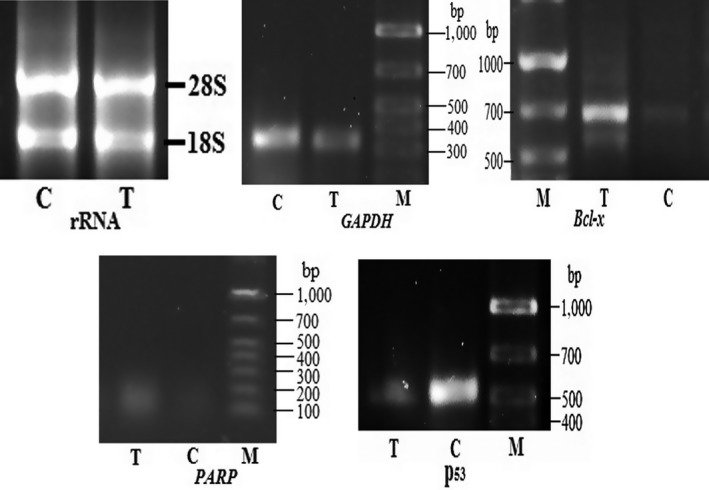
RNA extracted from both control and high‐dose carmoisine‐treated mouse and amplification of tumor‐related genes bcl‐X, PARP and p^53^ and control gene GAPDH. Total RNA was reverse‐transcribed using random hexamer, and PCR was performed with primers definite for Bcl‐x, PARP, and p^53^. PCR products separated on 1% agarose gel were stained with ethidium bromide. M, DNA ladder: T, RNA from high‐dose carmoisine fed mouse liver; C, RNA from control mouse liver

Clear amplification products of RT‐PCR were obtained by using GAPDH primers as control for carmoisine‐treated and carmoisine‐untreated RNAs. Then, the tumor‐related Bcl‐x, PARP, and p^53^ gene expressions were investigated. Amplification products for Bcl‐x, PARP and p^53^, respectively, are shown in Figure 2.

Higher level of expression for p^53^ gene was observed in control mouse, whereas the level of expression was significantly decreased in the carmoisine‐treated mouse. On the other hand, level of expression of Bcl‐x and PARP was increased in the carmoisine‐treated mouse where Bcl‐x was expressed as the isoform Bcl‐xL with the totally lost p^53^ gene expression as compared to the control mouse.

## 4 DISCUSSION

From the results of the toxicity study of the administration of carmoisine for 120 days, an impediment effect on growth rate was observed in all treatment groups of mice (Figure 1). Body weights were decreased significantly after 120 days in medium‐ and high‐dose groups of mice (Table 3) and were considered as primary indicator of the toxic effect of the dye. These data are in agreement with Amin et al. (2010) who showed that oral administration of high dose (100 mg/kg bw) of carmoisine decreased the body weight gain significantly in young male rats. These data are also in concord with the findings of Aboel‐Zahab et al. (1997) and Ford, Stevenson, and Evans (1987). Weight loss or reduced weight gain is considered by some authors to be a sign of toxicity (Ezeuko Vitalis, Nwokocha Chukwuemeka, Mounmbegna Philippe, & Nriagu Chinonso, 2007). A possible explanation for growth retardation of treatment groups of mice might be due to the disturbance effect of carmoisine dye that decreased the population of intestinal bacteria and hindered food absorption capacity of the intestinal surface. Chung et al. (1992) showed that azo dyes can be reduced by intestinal microbial azoreductase to aromatic amines, for example, sulfanilic acid, some of which is highly sensitizing and carcinogenic and hinders the digestion and absorption of food. In this study, lower feed efficacy was found among different groups of treatment mice although feed consumption rate was higher than control group (Table 4). This finding supported that digestion and absorption of diet might be inhibited due to administration of dye. Oral administration of dye at several levels suggested that it is poorly absorbed by the alimentary tract, with less than 3% appearing in the urine after 3 days (Lethco & Webb, 1966).

The findings of this study showed a significant decrease in Hb concentration of medium‐ and high‐dose group compared to control group (Table 5). These results support the observation of Aboel‐Zahab et al. (1997) who reported a decrease in RBCs and Hb of rat treated with chocolate colors A and B (sunset yellow, tartrazine, carmoisine, and brilliant blue) in varying concentration in diet. Basu and Kumar (2015) gave an explanation that this might be due to the binding of carmoisine with Hb, which results in a change in the secondary structure of Hb. The increased WBC counts from the CBC counts were observed in all treated groups in a dose‐dependent manner in this study. Borzelleca, Olson, and Reno (1989) explained that increase in WBC count may be due to acute inflammation for tumor development. The data from this study showed an increasing pattern of platelets in dose‐dependent manner, which might be due to the effect of secondary thrombocytosis. Platelets function as circulating cellular sensors that provide a unique link to immune responses and tissue repair (Nurden, 2011). Higher platelet count arises as a result of response of the immune system to infection, inflammation, tissue damage, and tumor. Here, monocytes were also increased in all treatment groups, but the exact mechanism and reasons for increasing have not been fully elucidated.

A significant increase in the activities of serum ALT, AST, and ALP was recorded in mice of medium‐ and high‐dose groups (Table 6). Similar results have been reported by Bansal, Bansal, Soni, and Bhatnagar (2005), and increased activities of ALT and AST are considered as a sensitive indicator of overall liver health and liver cell injury (Tameda et al., 2005). However, ALT is more precise for liver damage than AST. The elevation of serum aminotransferase activities might be due to liver, kidney, or heart tissue damage (Varely, Gowenlock, McMurray, & Mclauchlan, 1988) and increased permeability of cell membrane or synthesis, or decreased catabolism of aminotransferases (Malik, Singh, Gupta, Varman, & Paul, 1980). Westlake, Bunyan, Martin, Stanley, and Steed (1981) mentioned that the release of high levels of specific enzymes into bloodstream is dependent on both the degree and the type of damage exerted by the toxic compound administration. Webner (2003) showed in his study the abnormality in ALP level, which was caused by enzymes released from damaged liver. Same results were found in our experiment. Another study demonstrated that liver enzymes ALT, AST, and ALP were increased in rats that consumed diet with synthetic azo dues (Aboel‐Zahab et al., 1997; Amin et al., 2010; El‐Wahab & Moram, 2013; Helal, Zaahkouk, & Mekkawy, 2000; Mekkawy, Ali, & El‐Zawahry, 1998; Saxena & Sharma, 2014). Total bilirubin was increased significantly in high‐dose group as compared to control group, which is the results of hemolytic process and considered as impaired function of liver. This result is agreed with the results reported by El‐Wahab and Moram (2013) and Saxena and Sharma (2014).

A high concentration of serum TP, GLOB, URE, and CRE was found in high‐dose‐treated group of mice than control group (Table 7). These results are consistent with the findings of Aboel‐Zahab et al. (1997); Amin et al. (2010); and El‐Wahab and Moram (2013) who found the same effect on serum parameters in rats fed with synthetic dyes. AL‐Shinnawy (2009) explained that synthetic food dye stimulates protein biosynthesis, which in turn produces specific enzymes required for different physiological process and causes increase in serum protein concentration. He also reported that the elevated level of GLOB fraction leads toward the increase in immunoglobulin synthesis for the defense mechanism to protect the body from the toxic effects of synthetic food dyes. In previous studies, it was found that blood URE can be increased in all forms of kidney diseases (Varely et al., 1988). These changes reduced glomerular filtration rate as a result of an acute renal dysfunction. It is well known that serum level of URE and CRE depends on the glomerular filtration (Schramm et al., 1994).

A statistically significant reduction in total CHO level was found in medium‐ and high‐dose group of mice. These present results appear to support the previous study that oral consumption of azo dye tartrazine to male albino rats for 30 consecutive days causes reduction of serum CHO level by Amin et al. (2010), and these changes are considered as signs of liver disease (Singh, Khanna, & Singh, 1988). In contrast, Aboel‐Zahab et al. (1997) reported an increase in serum total CHO level in rats feeding with chocolate color. Brain weights were decreased in medium‐dose group of mice, whereas liver and kidneys weights were decreased in high‐dose group (Table 8), which is an important endpoint for identification of potentially harmful effects of chemicals (Bailey, Zidell, & Perry, 2004).

RNA was extracted from the liver tissue of different doses of mice to check the relative expression pattern of some tumor‐related genes. According to Basu and Kumar (2014), carmoisine can bind with DNA. The binding induced moderate conformational perturbations in the B‐form structure of DNA. Thus, it can exert its mutagenicity or carcinogenicity. In the present study, carmoisine‐induced progression of mutation and/or tumor cell was also confirmed by Bcl‐x, PARP, and p^53^ gene expression studies. Several genes in the bcl‐2 family have been identified, and this family is divided into a pro‐apoptotic class (e.g., Bax, Bak) and anti‐apoptotic class (e.g., Bcl‐2, Bcl‐xL). Bcl‐x gene product exists in two forms: Bcl‐xL (long), which blocks apoptosis in many systems, and the spliced Bcl‐xS (short), which acts as a dominant inhibitor of Bcl‐2 (Gradilone et al., 2003). Intensive gene expression of Bcl‐x was observed in treatment liver tissues, which indicated that apoptosis was blocked in those tissues, whereas it was significantly less in control liver tissues (Figure 2). Death of apoptotic cells is caused by a series of proteases termed caspases (Graf, Bode, & Häussinger, 2007; Madeo et al., 2009; Thornberry & Lazebnik, 1998). Caspases are activated during the apoptotic processes and can be categorized as initiator caspases (e.g., caspase‐8, caspase‐9, and caspase‐10) and effector caspases (e.g., caspase‐3, caspase‐6, and caspase‐7). Activation of initiator caspases, leading to the activation of executioner caspase‐3, activates DNA‐binding protein PARP‐1. PARP‐1 causes DNA to break down. As a result, apoptotic cell death occurs. In this study, deficient expression of DNA repair gene, PARP, was noticed in treatment liver tissues, confirming the blocking of apoptosis and induction of mutation or tumor in liver tissue. Generally, low level of expression of a DNA repair gene results in increased unrepaired DNA damages which, through replication errors, lead to mutations and/or tumor. Down‐regulation of PARP expression in control mice reveals that no alteration in DNA occurred. Furthermore, p^53^, known as tumor suppressor gene, was less down‐regulated in liver tissues of treated mice, whereas full expression was observed in control liver tissues, verifying the progression of mutation and/or tumor cell in treatment liver tissues.

## 5 CONCLUSION

In conclusion, although being approved for use in the food and pharmaceutical industries, synthetic food azo dyes like carmoisine can pose health risks. From results of the current study, it is evident that carmoisine can affect adversely and alter function of vital organs (e.g., liver and kidneys). Carmoisine not only increases the risk of hepatocellular damage at higher dose, but also induces cancer. It is also confirmed from this study that the liver is the major target organ of carmoisine toxicity. In our study, the safe level of this food color was low dose (4 mg/kg bw). Therefore, it is necessary to create public awareness regarding the negative health effects of synthetic food azo dyes and encourage consuming within ADI range.

## CONFLICT OF INTEREST

No conflict of interest to declare.

## ETHICAL CLEARANCE

This research work was approved by the Institutional Animal, Medical Ethics, Biosafety, and Biosecurity Committee (IAMEBBC) for Experimentations on Animal, Human, Microbes, and Living Natural Sources, memo no: 31/320/IAMEBBC/IBSC, Institute of Biological Sciences, University of Rajshahi, Bangladesh.

## INFORMED CONSENT

Written informed consent was obtained from all study participants.
